# The Metropolitan Sunday Fund: New Draft Laws of the Constitution

**Published:** 1913-05-24

**Authors:** 


### THE METROPOLITAN SUNDAY FUND.

### New Draft Laws of the Constitution.

At the Council at the Mansion House on the 19th instant the new draft laws of the constitution were submitted by the Special Committee to whom the matter had been referred. They were considered and finally approved for submission to the special meeting of constituents which will meet shortly under the presidency of the Lord Mayor on a date to be fixed to suit his lordship's convenience. The proposed new laws of the constitution will, we understand, be issued for the meeting of constituents. The Special Committee, consisting of the Rev. Prebendary Anderson, Sir John Bell, Sir William Church, the Rev. W. Hardy Harwood, and Sir C. Ernest Tritton, were specially thanked by the Council for the marked ability and judgment with which they have performed their difficult duties.

Should these new laws be approved, the constituents will henceforth consist of duly elected representatives. The congregations will be divided into three classes: (*a*) those who contribute £5 and under with one representative; (*b*) those who contribute from £5 to £30 with two representatives; and (*c*) those who contribute over £30 with three representatives. Power is given to the Council to invite any donor of not less than £20 to attend the annual meeting and to vote. The meetings of constituents will be summoned each year to receive and adopt the annual report and to elect a Council for the succeeding twelve months. Power is given to the Council to call special general meetings of the constituents, and each clergyman or minister contributing is, in future, to send to the secretary of the Fund the name and address of the representatives duly appointed by his congregation not later than June 30 in each year. Regulations are given as to the procedure at each meeting of constituents, and it is provided that all material resolutions adopted by the constituents should be in the form of recommendations to the Council, by whom they will be considered, and the result of such consideration reported to a special meeting of the constituents. The rules governing the work of the committee of distribution and the methods of distribution have been revised. The new constitution, when adopted, will strengthen the hands of the constituents by providing that one-fifth of the members of the Council shall retire annually in rotation, and a list of their names, together with any new names nominated, will be submitted for election at the annual meeting. This meeting is fixed to be held in the month of December in each year and each new Council will be charged with the duty of electing the committee of distribution. By these regulations the constituents should possess power to nominate new members of Council and full representation in the management of the affairs of the Hospital Sunday Fund.

